# Three-Dimensional visualization reconstruction assisted in the treatment of retroperitoneal liposarcoma: a case report

**DOI:** 10.3389/fsurg.2025.1609274

**Published:** 2025-07-16

**Authors:** Xiangxiang Ren, Tianhao Xie, Litao Liu, Xiaoshi Jin, Meng Zhang

**Affiliations:** ^1^ Department of General Surgery, Affiliated Hospital of Hebei University, Baoding, Hebei, China; ^2^ Department of Dermatology, Affiliated Hospital of Hebei University, Baoding, Hebei, China

**Keywords:** three-dimensional visualization reconstruction, retroperitoneal liposarcoma, surgical precision, preoperative planning, case report

## Abstract

**Objective:**

To evaluate the clinical efficacy of three-dimensional visualization reconstruction (3DVR) in surgical planning for complex retroperitoneal liposarcoma (RLS), we report a case of a 64-year-old female patient presenting with a mass posterior to the spleen. CT/MRI revealed a retro-splenic mass, later pathologically confirmed as dedifferentiated liposarcoma. 3D reconstruction precisely delineated a 12 × 6.0 cm tumor with three splenic feeding vessels, enabling preoperative simulation of an *en bloc* resection combined with splenectomy. Gross total resection (R0 resection) was achieved, and pathology confirmed no splenic invasion. No recurrence was observed during 6-month follow-up.

**Conclusion:**

Compared to 2D imaging, 3D reconstruction improved stereoscopic assessment of tumor-vessel-organ relationships, reducing intraoperative uncertainty. Challenges in retroperitoneal soft-tissue contrast were mitigated using advanced segmentation. The technique enhances surgical precision, lowers operative risks, and may improve recurrence-free survival. Integration with virtual reality could further optimize preoperative planning, advocating its adoption in complex abdominal oncology.

## Introduction

Retroperitoneal liposarcoma (RLS), a rare but aggressive mesenchymal tumor, poses significant therapeutic challenges due to its anatomical complexity, late clinical presentation, and propensity for local recurrence (1) Complete macroscopic resection remains the cornerstone of curative intent, yet achieving negative margins (R0) is often hindered by the tumor's intricate relationships with retroperitoneal vasculature and viscera (2). Conventional imaging modalities, such as two-dimensional (2D) computed tomography (CT), provide limited spatial resolution for preoperative planning, particularly in cases of distorted anatomy or multifocal vascular involvement (3).

## Case presentation

**Figure 1:**
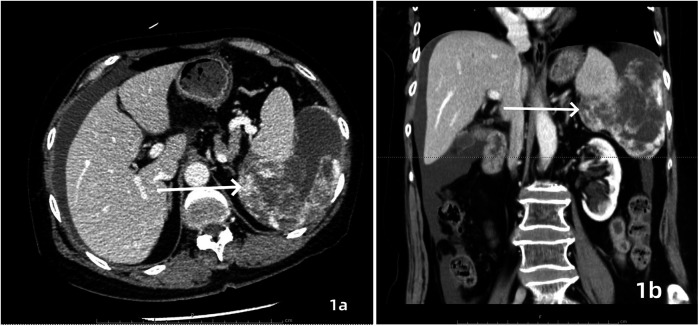
Contrast-enhanced CT, axial **(a)** and coronal **(b)** views demonstrated an irregularly contoured mass posterior to the spleen with heterogeneous enhancement. Focal hyperdense areas (white arrows) suggesting intratumoral hemorrhage or calcification were observed within the lesion.

**Figure 2:**
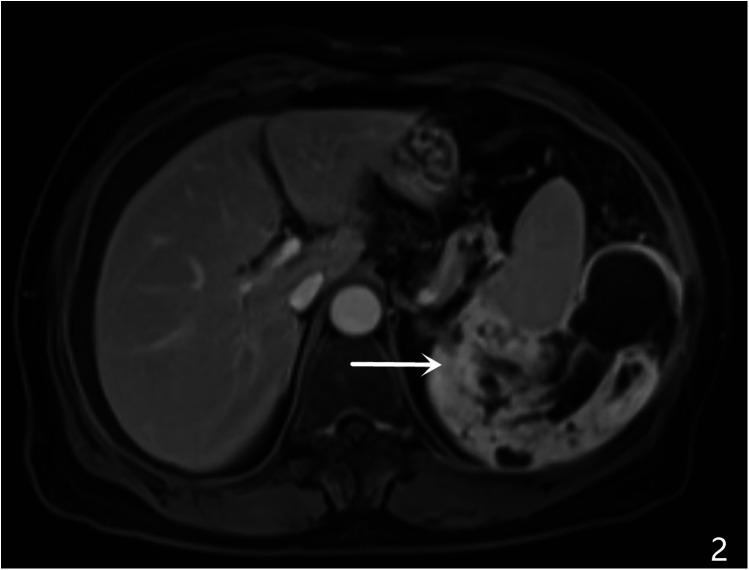
MRI characteristics, A large heterogenous mass was identified in the left retro-splenic region, showing: hypointense signal on T1-weighted imaging (T1WI) hyperintense signal on T2-weighted imaging (T2WI) multiple cystic components with fluid-fluid levels (white arrows) diffusion-weighted imaging (DWI) revealed restricted diffusion in solid components (ADC value: 0.89 × 10^−3^ mm²/s). Post-contrast sequences displayed heterogeneous enhancement with ill-defined splenic interface.

**Figure 3:**
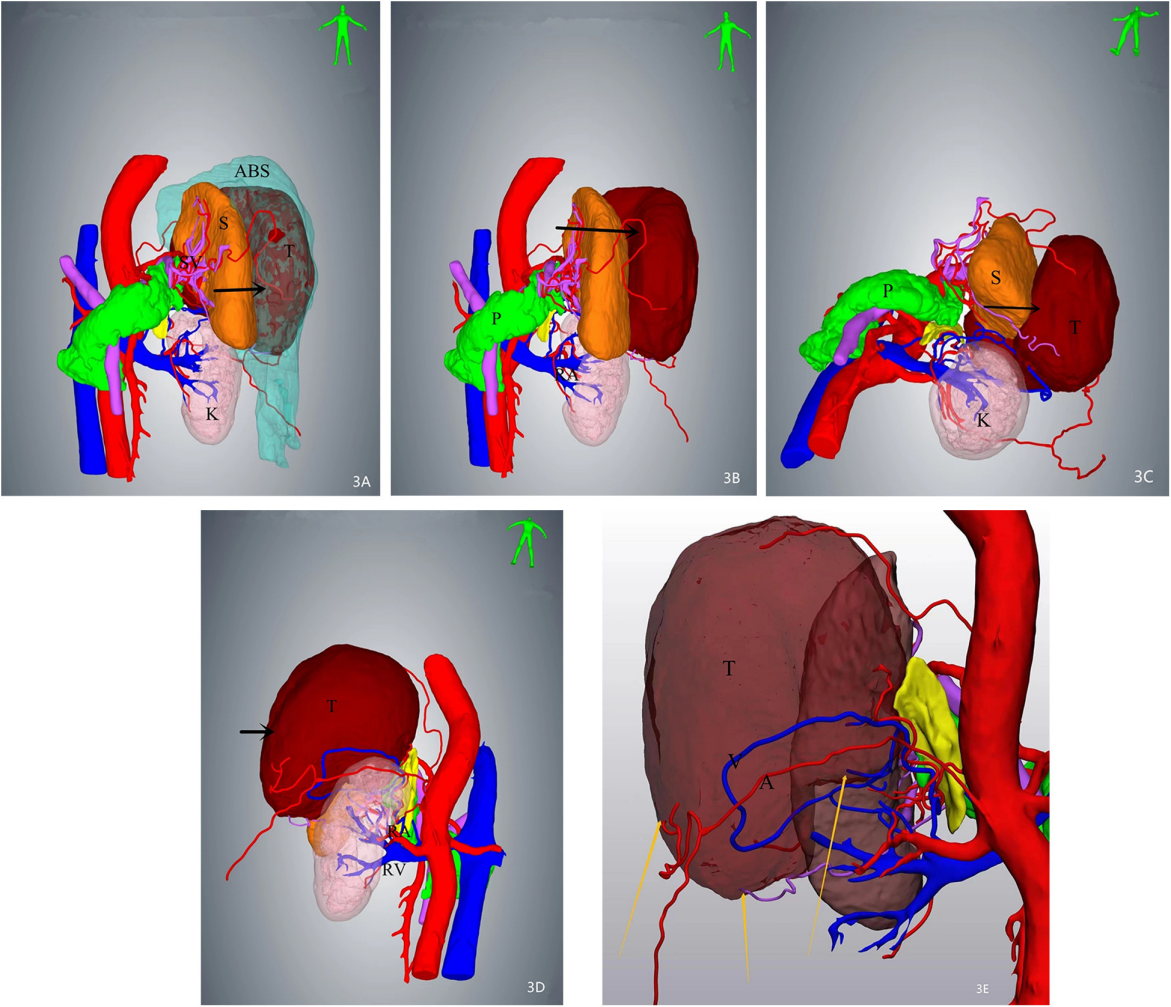
Three-dimensional (3D) reconstruction localizes the mass (arrows) posterior to the spleen, demonstrating intimate anatomical adjacency. Three feeding vessels are identified along the tumor periphery. **(A)** Superior view of the mass with associated ascites. **(B)** Tumor localization after digital removal of ascites. **(C)** Inferior-to-superior view illustrating spatial relationships. **(D)** Dorsal perspective highlighting tumor proximity to retroperitoneal structures. **(E)** Arteries and veins of the mass were visible.

A 64-year-old female patient was admitted with a 5-month history of intermittent abdominal discomfort. Physical examination revealed mild abdominal distension with slight tenderness in the left upper quadrant, without rebound tenderness or muscle rigidity. No palpable mass was detected. Contrast-enhanced abdominal CT (Figures 1a,b) demonstrated a suspicious malignant lesion lateral to the spleen, possibly of splenic origin. Abdominal MRI (Figure 2) revealed: (1) a retro-splenic mass suggestive of liposarcoma; (2) hemoperitoneum. The clinical diagnosis was retroperitoneal liposarcoma. Preoperative Three-Dimensional Reconstruction Preoperative three-dimensional reconstruction (Figures 3A–E) localized the tumor dorsal to the spleen, showing three vascular branches originating from the spleen feeding the tumor. Clear boundaries were observed between the tumor and adjacent structures (kidney, renal artery, pancreas, and bowel), with significant peritumoral fluid accumulation.

**Figure 4:**
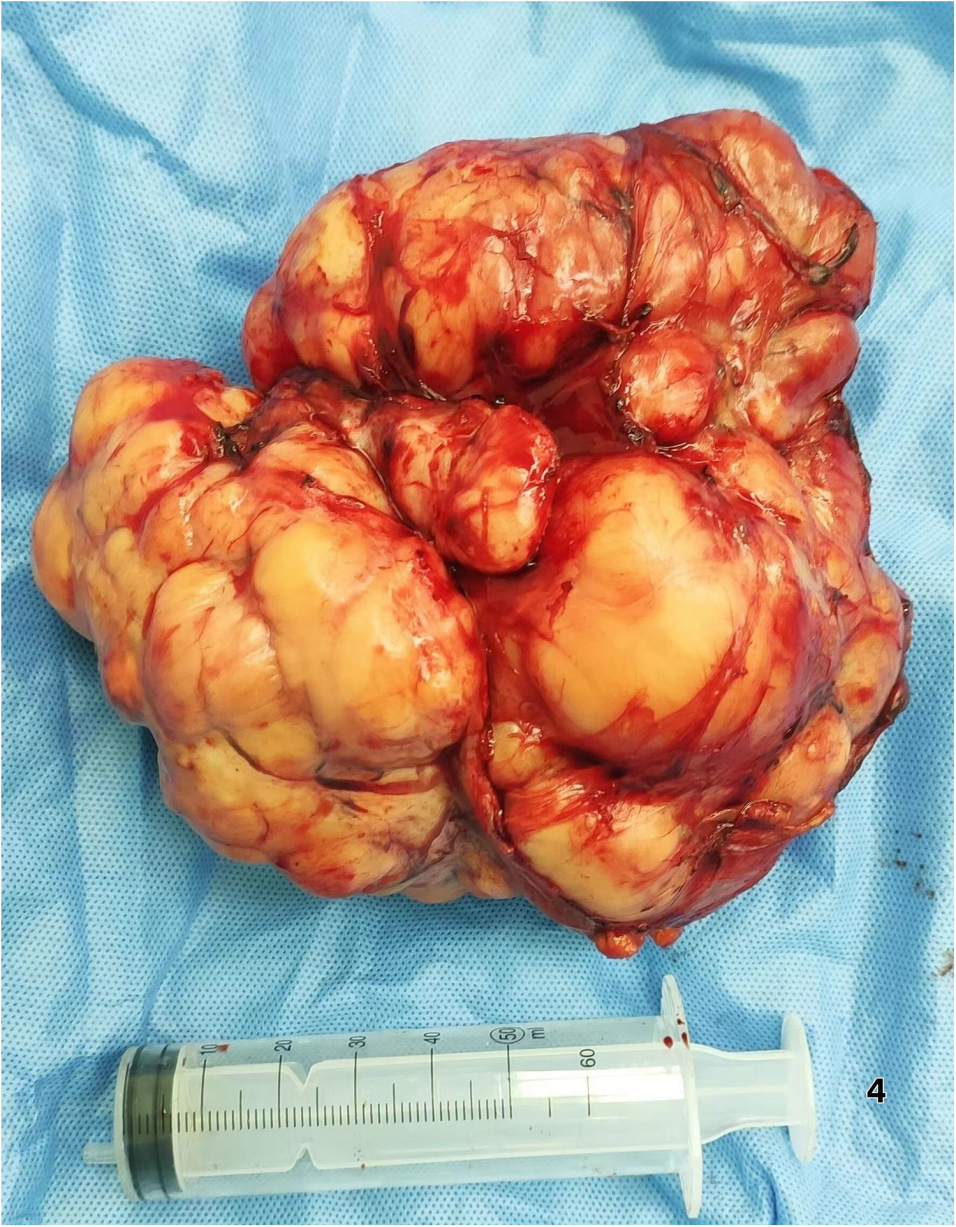
Demonstrates the *en bloc* resected tumor, showing a clear boundary and an intact capsule.

Surgical Intervention Exploratory laparotomy identified a 12 cm × 6.0 cm × 5 cm encapsulated mass (Figure 4) in the left retro-splenic region. The mass exhibited dense adhesions to the spleen, with partial blood supply derived from splenic vessels. The total operative time was 185 min. Estimated intraoperative blood loss was 450 ml, requiring no blood transfusion. Due to challenging dissection and significant intraoperative bleeding from tumor-splenic adhesions, *en bloc* resection of the tumor with splenectomy was performed. The patient was transferred to the general ward postoperatively without ICU admission.

**Figure 5:**
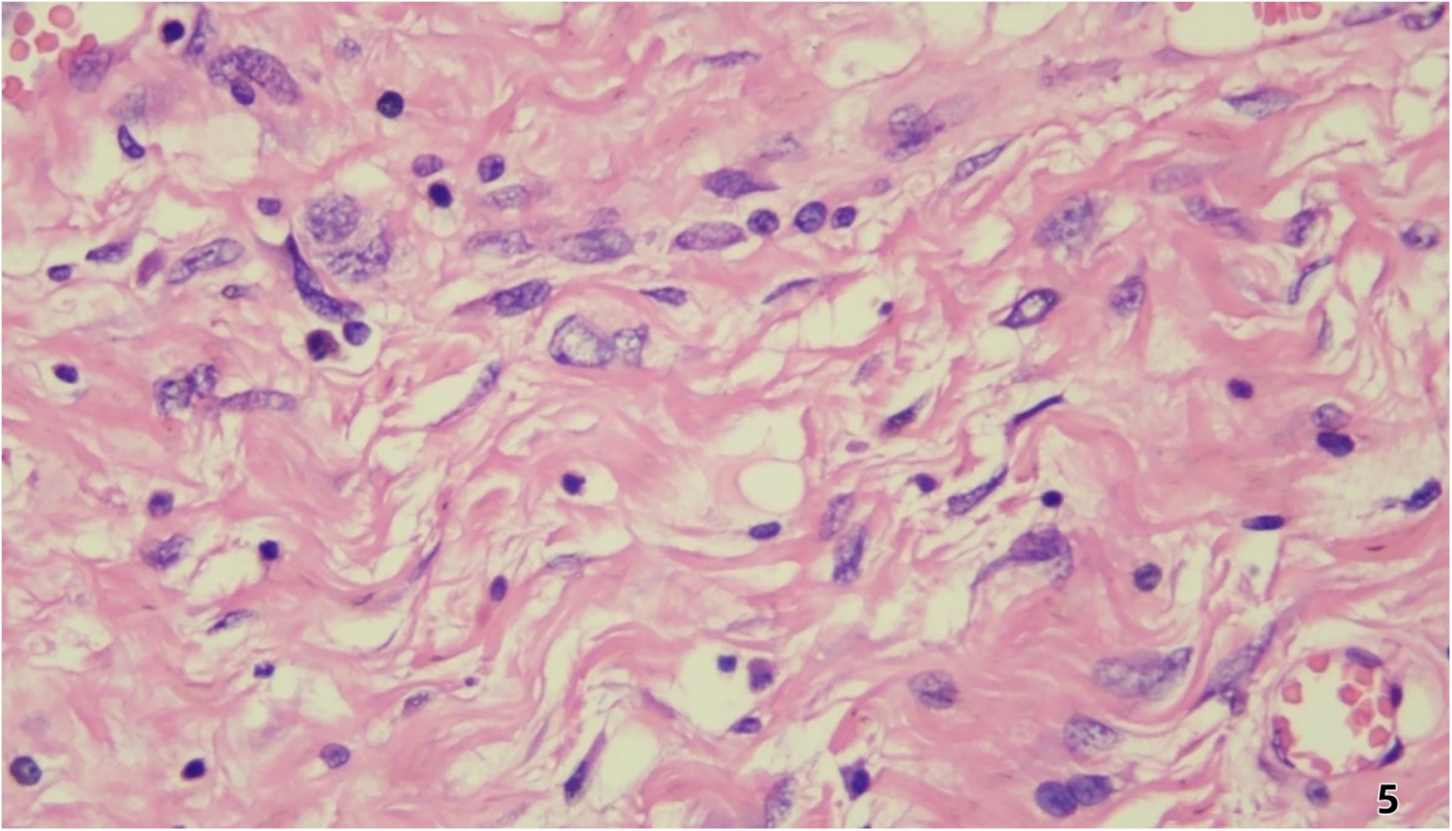
Histopathological examination revealed predominantly spindled to polygonal cells exhibiting enlarged hyperchromatic nuclei with prominent nucleoli and readily identified mitotic figures. The neoplastic cells displayed disorganized architectural patterns, including fascicular, storiform, or diffuse arrangements, within a stromal background demonstrating variable myxoid change, hyalinization, or collagenization.

Pathological and Immunohistochemical Findings Histopathological examination (Figure 5) revealed predominantly spindled to polygonal cells exhibiting enlarged hyperchromatic nuclei with prominent nucleoli and readily identified mitotic figures. The neoplastic cells displayed disorganized architectural patterns, including fascicular, storiform, or diffuse arrangements, within a stromal background demonstrating variable myxoid change, hyalinization, or collagenization. Immunohistochemistry showed: Cyclin-dependent kinase 4 (CDK4) (+)、Mouse double minute 2 homolog (MDM2) (+)、Ki-67 (10%+)、CD34 (vascular+)、ERG (vascular+)、CK (-)、EpCAM (-)、CD68 (focal+)、Calretinin (-)、p16 (focal+).

Postoperative Follow-up The patient resumed oral intake on postoperative day 2 and was discharged on day 7 without complications (Clavien-Dindo grade 0). At the 6-month surveillance (contrast-enhanced CT), no recurrence was observed.

## Discussion

The management of retroperitoneal liposarcoma (RLS) hinges on achieving complete macroscopic resection, as incomplete tumor removal remains the primary driver of postoperative recurrence (1, 2). This challenge is compounded by the tumor's insidious growth within anatomically complex retroperitoneal spaces, where lesions often exceed 20 cm at diagnosis, distorting normal anatomy and obscuring critical vascular landmarks (3). While contrast-enhanced CT provides foundational diagnostic insights (3), its inherent 2D limitations—poor spatial resolution of tumor-vessel interfaces and inadequate soft-tissue contrast—frequently undermine preoperative risk stratification (4).

This case exemplifies how 3D visualization reconstruction addresses these limitations. By converting 2D CT datasets into interactive stereoscopic models, surgeons gain un-paralleled spatial perception of tumor morphology, vascular supply (e.g., three splenic feeding vessels in this case), and anatomical relationships (5). Dynamic manipulation of these models—rotation, magnification, and virtual tissue dissection—enables pre-operative simulation of resection margins and contingency planning for vascular control (6, 7). Such capabilities proved pivotal in our patient, where 3D guidance facilitated *en bloc* resection of a 12 cm splenic-adherent mass while minimizing intraoperative exploration and hemorrhage. Our estimated blood loss (EBL) of 450 ml was significantly lower than that reported for typical RLS cohorts (600–1,200 ml) (3, 8). This reduced EBL is attributable to preoperative 3D planning, which identified splenic feeding vessels and enabled proactive vascular control. Operative time (185 min) was within the lower range of published data (1, 3), likely due to minimized intraoperative exploration. The absence of transfusions or complications contrasts sharply with the higher rates reported in the literature (1, 2, 5), supporting 3DVR's role in mitigating surgical morbidity. The gross specimen of the postoperative tumor (Figure 4) demonstrated well-defined borders, consistent with the 3D computational model predictions.

Notably, 3D technology's adoption in retroperitoneal oncology lags behind its success in solid organ surgery [e.g., hepatic/pancreatic resections (9)]. This disparity stems from technical barriers: retroperitoneal fat's homogeneous density on CT complicates tumor segmentation, while multifocal vascular encasement demands advanced algorithms to differentiate tumor margins from displaced structures (8). However, emerging solutions—such as dual-energy CT for enhanced soft-tissue contrast and AI-driven edge detection—are bridging these gaps, promising higher-fidelity 3D models (10).

Current evidence (11) confirms that complete surgical resection remains the cornerstone therapeutic intervention for retroperitoneal liposarcoma, demonstrating significant reduction in local recurrence rates compared to incomplete excision. The integration of three-dimensional visualization reconstruction technology enables precise intraoperative tumor demarcation, which critically facilitates R0 resection achievement and is associated with statistically significant reduction in locoregional recurrence rates. The high recurrence rates of RLS [40%–60% within 5 years (10)] further underscore the need for precision tools. Although the current 6-month follow-up interval remains limited in duration for recurrence assessment, the patient will be enrolled in a structured surveillance protocol comprising annual contrast-enhanced MRI (per NCCN Guidelines Version 3.2023) over a minimum 5-year continuum, with protocol-defined interim analyses to be reported at standardized 24-month intervals using RECIST 1.1 criteria. Reoperative resection, though fraught with complications, remains the sole curative option for recurrent disease. Here, 3D reconstruction's value extends beyond initial surgery: in reoperative fields with scarred anatomy, it provides a virtual roadmap to distinguish tumor pseudocapsules from adhesions, potentially reducing iatrogenic injuries (12).

Future Directions: Integrating 3D models with virtual reality (VR) platforms could enable immersive preoperative rehearsals, while machine learning may predict tumor biology to optimize resection margins. For now, this case validates 3D reconstruction as a transformative adjunct in RLS management, advocating its routine integration into multidisciplinary workflows to mitigate surgical morbidity and improve oncologic outcomes.

## Conclusions

3D reconstruction technology significantly enhances retroperitoneal tumor management by enabling precise anatomical mapping and complete resection, thereby reducing recurrence risks. Future integration with virtual reality (VR) will allow immersive surgical simulation, optimizing strategies for complex anatomy. Broader adoption of 3D-guided workflows promises to redefine standards of care, improving survival outcomes in retroperitoneal malignancies. While our technique achieved R0 resection in this cohort—a known predictor of reduced recurrence—longer follow-up is needed to assess its survival impact.

## Data Availability

The original contributions presented in the study are included in the article/Supplementary Material, further inquiries can be directed to the corresponding author.
